# Supplementary material for: Unraveling the Molecular Basis of the Dystrophic Process in Limb-Girdle Muscular Dystrophy LGMD-R12 by Differential Gene Expression Profiles in Diseased and Healthy Muscles
Source: Cells. 2022 Apr 30;11(9):1508. doi: 10.3390/cells11091508 (PMC9104122; doi:10.3390/cells11091508)
Supplement: Supplementary file 1 [file cells-11-01508-s001.zip › cells-1676651-supplementary.pdf]

Table S1. Overview of included samples and quality control in patients.

Patient	Muscle	Quality control (QC) passed	Reads
1	rectus femoris	Passed QC	20948322
1	semimembranosus	Passed QC	22412762
1	vastus lateralis	Low count and mapping quality	19787265
2	rectus femoris	Passed QC	21569193
2	semimembranosus	Passed QC	18574761
2	vastus lateralis	Passed QC	18284915
3	rectus femoris	Passed QC	22547247
3	semimembranosus	Adapters and duplicated reads (*)	2728665
3	vastus lateralis	Passed QC	22401618
4	rectus femoris	Passed QC	20995031
4	semimembranosus	Passed QC	23652756
4	vastus lateralis	Passed QC	18748284
5	rectus femoris	Passed QC	20962680
5	semimembranosus	Adapters and duplicated reads (*)	241860
5	vastus lateralis	Adapters and duplicated reads (*)	704789
6	rectus femoris	Passed QC	16255225
6	vastus lateralis	Low count and mapping quality	24860024
7	rectus femoris	Passed QC	15329355
7	vastus lateralis	Passed QC	16513759
8	rectus femoris	Passed QC	23206339
8	semimembranosus	Passed QC	21883046
9	rectus femoris	Passed QC	22539668
9	semimembranosus	Passed QC	13255519
9	vastus lateralis	Passed QC	16523750
10	rectus femoris	Passed QC	22682550
10	semimembranosus	Low count and mapping quality	30346007
10	vastus lateralis	Passed QC	25162767
11	rectus femoris	Low count and mapping quality	15940634
11	semimembranosus	Passed QC	19356319
11	vastus lateralis	Passed QC	22379794
12	semimembranosus	Adapters and duplicated reads (*)	1064131

12	vastus lateralis	Adapters and duplicated reads (*)	190562
13	rectus femoris	Passed QC	25768066
13	semimembranosus	Passed QC	22458508
13	vastus lateralis	Passed QC	18122838
14	rectus femoris	Passed QC	22867758
14	semimembranosus	Passed QC	16273735
14	vastus lateralis	Passed QC	21394915
15	rectus femoris	Passed QC	22206329
15	semimembranosus	Passed QC	2521847
15	vastus lateralis	Passed QC	16159284

(*) Five samples were excluded after quality filtering for read mapping (see Methods section).

Table S2. Overview of included samples and quality control in control individuals.

Control	Muscle	Quality control (QC) passed	Reads
C1	rectus femoris	Passed QC	22343420
C1	semimembranosus	Passed QC	18971022
C1	vastus lateralis	Passed QC	19270537
C2	rectus femoris	Passed QC	16472959
C2	semimembranosus	Passed QC	13610135
C2	vastus lateralis	Passed QC	9618857
C3	rectus femoris	Passed QC	18505267
C3	semimembranosus	Passed QC	20112255
C3	vastus lateralis	Passed QC	18611807
C4	rectus femoris	Passed QC	53580100
C4	semimembranosus	Passed QC	15698877
C4	vastus lateralis	Passed QC	19462360
C5	rectus femoris	Passed QC	21298875
C5	semimembranosus	Passed QC	20265084
C5	vastus lateralis	Passed QC	21811325
C6	rectus femoris	Passed QC	14783681
C6	semimembranosus	Low count and mapping quality	15009861
C7	rectus femoris	Passed QC	20028735
C7	semimembranosus	Passed QC	22462082
C7	vastus lateralis	Passed QC	18850415
C8	rectus femoris	Passed QC	22003947
C8	vastus lateralis	Passed QC	17693870
C9	rectus femoris	Passed QC	22134917
C9	semimembranosus	Low count and mapping quality	7486932
C9	vastus lateralis	Passed QC	14089411
C10	rectus femoris	Passed QC	16174715
C10	semimembranosus	Passed QC	18043701

C10	vastus lateralis	Passed QC	22100926
C11	rectus femoris	Passed QC	16632859
C11	semimembranosus	Passed QC	21779784
C11	vastus lateralis	Passed QC	19711576
C12	rectus femoris	Passed QC	21244333
C12	semimembranosus	Passed QC	18513026
C12	vastus lateralis	Passed QC	16754138
C13	rectus femoris	Passed QC	19239712
C13	semimembranosus	Passed QC	20681769
C13	vastus lateralis	Passed QC	21306295
C14	rectus femoris	Passed QC	18902470
C14	semimembranosus	Passed QC	20825095
C14	vastus lateralis	Passed QC	18165638
C15	rectus femoris	Passed QC	21567960
C15	semimembranosus	Passed QC	22082492
C15	vastus lateralis	Passed QC	21049516

Table S3. Top 50 of most differentially expressed genes across early stage patient muscles.

**Marker Gene Analysis Across Patient Muscles**			
**Rank**	**Semimembranosus**	**Rectus Femoris**	**Vastus Lateralis**
1	COMP	FEZF1-AS1	LINC01854
2	HAND2	LINC02107	P2RX3
3	HOXD9	PVALB	CHAC1
4	HAND2-AS1	C1orf158	SNCB
5	SPP1	CDH22	HOXA13
6	HOXD-AS2	LRRC37A7P	TYRP1
7	SAA1	LINC01773	KCTD8
8	SAA2	LINC02119	SPAG17
9	GBP6	PLCH1	NEU4
10	PLA2G2A	MYH1	ZNF750
11	AQP6	ATRNL1	SIM2
12	CCL18	GREM2	TBX5-AS1
13	MYBPH	GRID2	PPBP
14	COL20A1	PDE4DIPP1	LAMC3
15	TNC	ENSAP2	LINC01018
16	SFTA1P	HCN1	CRYM
17	MYBL2	ERBB4	RSPO3
18	CHI3L1	CSMD1	HMGCS2
19	S100A3	ZFPM2-AS1	IGSF21
20	FRMD1	PRR32	IL20RA
21	ACTC1	RHOXF1-AS1	HOXC12
22	TSPEAR	FAM184B	IGHD
23	USH2A	CSPG5	GDNF
24	TYMS	CALML6	LCA5L
25	FHAD1	MKRN3	FGF22
26	MYH3	CHGB	GAS2

27	COL22A1	RYR2	MAPT-AS1
28	SCUBE1	UGT3A1	SLC26A9
29	CD180	GRIA2	ADAMTS19-AS1
30	MYEOV	TCF24	HCN2
31	HMGN1P4	PODNL1	RBM22P1
32	TMEM132E	MYL10	FZD9
33	MORN3	MYLK4	PCDH8
34	HOXD8	RPS4XP14	FAM163A
35	C10orf67	OPRD1	LINC01968
36	CACNA1F	UNC13C	SLED1
37	ITGB2-AS1	LINC01886	FLT1P1
38	AMZ1	B3GALT1	SLC51A
39	LRRC74A	FABP7	FKBP4P6
40	PMEL	RNU6-144P	SNRPFP1
41	HDAC1P1	NDUFA5P11	GBX2
42	KIF18B	ACTN3	KLHDC7B
43	HYDIN	LRRC3B	RPL29P11
44	FOSL1	ADGRF4	PRSS12
45	TGFA	TACR1	TBX5
46	KRT7	RN7SL336P	MTND4P24
47	RNF32	MMP27	MROH5
48	CACNA1I	CTNNA2	ABHD1
49	SIGLECL1	NANOS1	SLC38A5
50	STAB2	CCDC192	BDNF

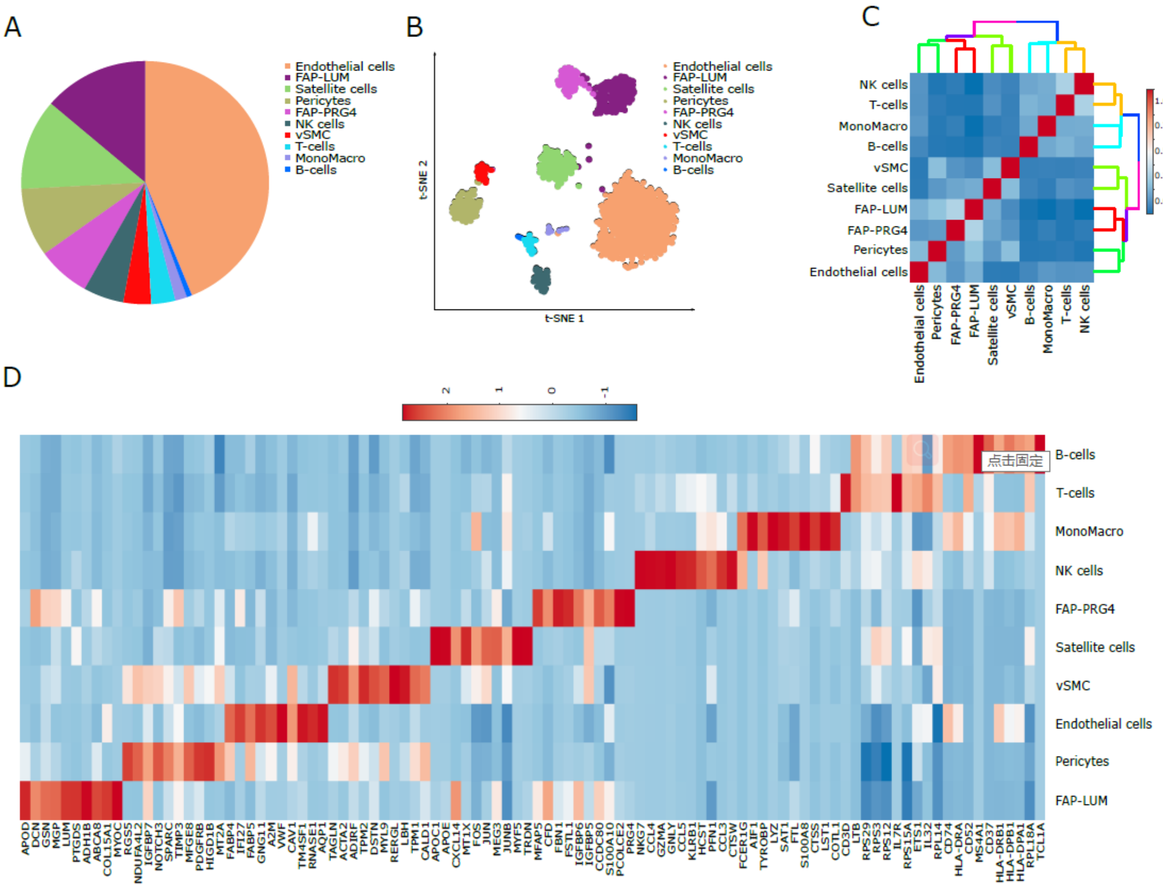

Figure S1. Re-analysis of scRNA-seq data. (**A**) Pie chart showing the cell type composition of the re-analyzed muscle single cell scRNA-seq data. (**B**) t-SNE analysis of the re-analyzed muscle scRNA-seq data, color coded for cell type. (**C**) Dendrogram visualization of hierarchical clustering and heatmap analysis on gene signature correlations between cell types. (**D**) Heatmap analysis of the top 10 marker genes of all cell types in the re-analyzed muscle scRNA-seq data. Abbreviations: vascular smooth muscle cells (vSMC), natural killer cells (NK).

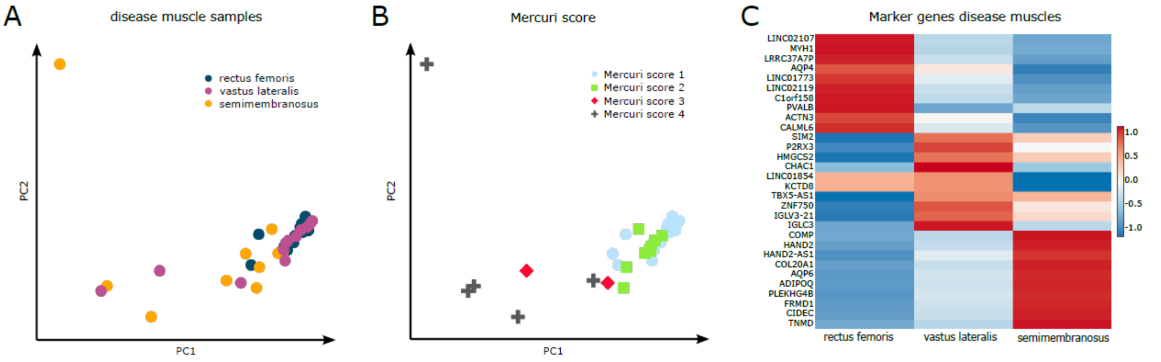

Figure S2. Analysis of patient samples. (**A**) PCA plot of disease samples color coded for muscle of origin. Note that in contrast to healthy samples (see Figure 4a), disease samples do not group according to muscle. (**B**) PCA plot of disease samples color coded for Mercuri score. Note that disease samples group according to disease stage rather than muscle. (**C**) Heatmap analysis of the top 10 marker genes of all disease muscles.
